# 2-Bromo-1-[1-(4-bromo­phen­yl)-5-methyl-1*H*-1,2,3-triazol-4-yl]ethanone

**DOI:** 10.1107/S1600536814014603

**Published:** 2014-06-25

**Authors:** Alexander S. Bunev, Marina A. Troshina, Gennady I. Ostapenko, Andzhela P. Pavlova, Victor N. Khrustalev

**Affiliations:** a Department of Chemistry and Chemical Technology, Togliatti State University, 14 Belorusskaya St, Togliatti 445667, Russian Federation; b Department of General and Theoretical Physics, Togliatti State University, 14 Belorusskaya St, Togliatti 445667, Russian Federation; c X-Ray Structural Centre, A.N. Nesmeyanov Institute of Organoelement Compounds, Russian Academy of Sciences, 28 Vavilov Street, B-334, Moscow 119991, Russian Federation

**Keywords:** crystal structure

## Abstract

The asymmetric unit of the title compound, C_11_H_9_Br_2_N_3_O, contains two crystallographically independent mol­ecules with similar geometries; the Br—C—C=O torsion angles are 1.2 (4) and −2.8 (4)°, and the benzene and triazole rings are inclined o one another by 51.90 (16) and 51.88 (16)°. The two molecules are related by a pseudo-screw 2_1_ axis directed along [100]. In the crystal, mol­ecules are linked into a three-dimensional network by weak C—H⋯O and C—H⋯N hydrogen bonds and secondary Br⋯Br [3.5991 (8) and 3.6503 (9) Å] inter­actions.

## Related literature   

For applications of 1,2,3-triazoles, see recent reviews by Agalave *et al.* (2011 ▶); Thirumurugan *et al.* (2013 ▶). For the crystal structures of related compounds, see: Danence *et al.* (2011 ▶); Zeghada *et al.* (2011 ▶); Abdel-Wahab, Abdel-Latif *et al.* (2013 ▶); Abdel-Wahab, Mohamed *et al.* (2013 ▶).
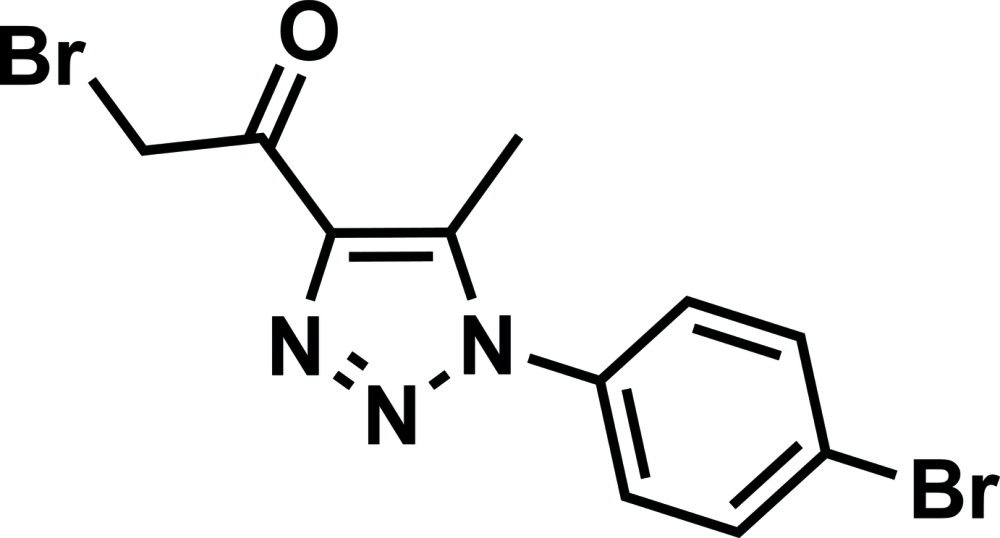


## Experimental   

### 

#### Crystal data  

C_11_H_9_Br_2_N_3_O
*M* *_r_* = 359.03
Monoclinic, 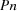

*a* = 3.9699 (10) Å
*b* = 19.437 (5) Å
*c* = 15.402 (4) Å
β = 90.908 (3)°
*V* = 1188.3 (5) Å^3^
*Z* = 4
Mo *K*α radiation
μ = 6.81 mm^−1^
*T* = 100 K
0.30 × 0.03 × 0.03 mm

#### Data collection  

Bruker APEXII CCD diffractometer
Absorption correction: multi-scan (*SADABS*; Bruker, 2003 ▶) *T*_min_ = 0.235, *T*_max_ = 0.822
18272 measured reflections
6924 independent reflections
6426 reflections with *I* > 2σ(*I*)
*R*_int_ = 0.028

#### Refinement  

*R*[*F* ^2^ > 2σ(*F* ^2^)] = 0.028
*wR*(*F* ^2^) = 0.067
*S* = 1.04
6924 reflections
309 parameters
2 restraints
H-atom parameters constrained
Δρ_max_ = 0.72 e Å^−3^
Δρ_min_ = −0.59 e Å^−3^
Absolute structure: Flack (1983 ▶), 3428 Friedel pairs
Absolute structure parameter: 0.032 (7)

### 

Data collection: *APEX2* (Bruker, 2005 ▶); cell refinement: *SAINT* (Bruker, 2001 ▶); data reduction: *SAINT*; program(s) used to solve structure: *SHELXTL* (Sheldrick, 2008 ▶); program(s) used to refine structure: *SHELXTL*; molecular graphics: *SHELXTL*; software used to prepare material for publication: *SHELXTL*.

## Supplementary Material

Crystal structure: contains datablock(s) global, I. DOI: 10.1107/S1600536814014603/cv5467sup1.cif

Structure factors: contains datablock(s) I. DOI: 10.1107/S1600536814014603/cv5467Isup2.hkl

Supporting information file. DOI: 10.1107/S1600536814014603/cv5467Isup3.cml

CCDC reference: 1009335

Additional supporting information:  crystallographic information; 3D view; checkCIF report

## Figures and Tables

**Table 1:** Hydrogen-bond geometry (Å, °)

*D*—H⋯*A*	*D*—H	H⋯*A*	*D*⋯*A*	*D*—H⋯*A*
C10—H10⋯O1^i^	0.95	2.44	3.205 (4)	137
C13—H13*B*⋯N6^ii^	0.99	2.49	3.467 (4)	170
C21—H21⋯O2^iii^	0.95	2.45	3.210 (4)	137
C24—H24*B*⋯N3	0.99	2.49	3.482 (4)	177

Symmetry codes: (i) 
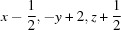
; (ii) 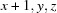; (iii) 
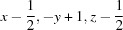
.

**Fig. 1.**
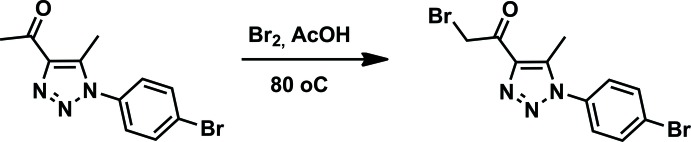
The synthesis of 7-nitro-2-phenylimidazo[2,1-b][1,3]benzothiazole.

**Fig. 2.**
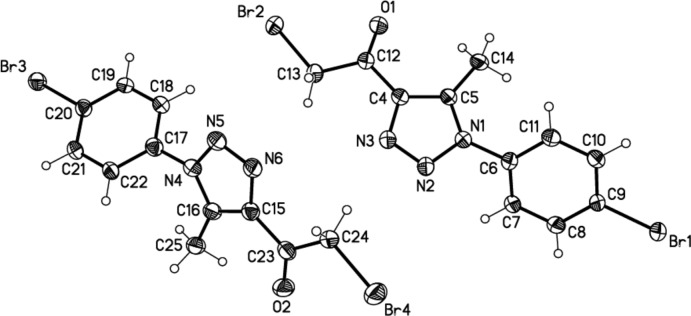
Two independent molecules in the asymmetric unit of I. Displacement ellipsoids are presented at the 50% probability level. H atoms are depicted as small spheres of arbitrary radius.

**Fig. 3.**
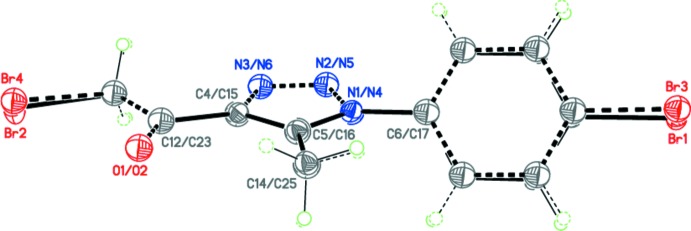
Superposition of the two independent molecules of I.

## supplementary crystallographic information

### S1. Comment

1-*H*-1,2,3-Triazole occupies a special place in heterocyclic chemistry because it is the core structure of many agents of various interests (used as pharmaceuticals, afrochemicals *etc.*). These compounds and their derivatives demonstrate antiviral, antimicrobial, anticancer activities as well as the inhibition activity of VIM-2 Metallo-β-Lactamase, and α-Glucosidases (Agalave *et al.*, 2011; Thirumurugan *et al.*, 2013). In this work, the title compound was prepared by the reaction of 1-(1-(4-bromophenyl)-5-methyl-1*H*-1,2,3-triazol-4-yl)ethanone with bromine (Figure 1), and its structure was unambiguously established by the X-ray diffraction study.

The title compound (I) crystallizes in the non-centrosymmetric monoclinic space group *P*n with two crystallographically independent molecules in the asymmetric unit (Figure 2). The two crystallographically independent molecules are related by *pseudo*-screw axis 2_1_ directed in [100] and, consequently, have very similar geometries (Figure 3). The position of the *pseudo*-screw axis 2_1_ with the approximate coordinates of (*x*, 0.75, 0.55) is shifted relative to the crystallographic position of (*x*, 0.75, 0.75) by *ca.* 0.20 Å towards the *c* axis, apparently, due to the formation of the more dense crystal packing as well as different non-valent intermolecular interactions.

The bond lengths and angles within the molecules of I are in a good agreement with those found in the related compounds (Danence *et al.* 2011; Zeghada *et al.* 2011; Abdel-Wahab, Abdel-Latif *et al.* 2013; Abdel-Wahab, Mohamed *et al.* 2013). The 2-bromo-1-ethanone substituent in the molecules of I has a significantly flattened conformation (the Br–C–C═O torsion angles are 1.2 (4) and -2.8 (4)° for the two independent molecules, respectively), with the carbonyl group directed toward the methyl substituent, and lies almost within the triazole plane (r.m.s. deviations are 0.037 and 0.023 Å for the two independent molecules, respectively) (Figure 2). The bromo-benzene substituent is twisted by 52.30 (6) and 51.81 (6)° (for the two independent molecules, respectively) relative to this main plane of the molecule.

In the crystal, the molecules of I are linked into three-dimensional framework by intermolecular C–H···O and C–H···N hydrogen bonds (Table 1) as well as secondary Br1···Br2^i^ (3.5991 (8) Å) and Br3···Br4^ii^ (3.6503 (9) Å) interactions [symmetry codes: (i) *x*, *y*, *z*+1; (ii) *x*, *y*, *z*-1].

### S2. Experimental

Bromine (1.6 g, 10 mmol) was slowly added to a solution of 1-(1-(4-bromophenyl)-5-methyl-1*H*-1,2,3-triazol-4-yl)ethanone (2.8 g, 10 mmol) in AcOH (30 mL). The mixture was stirred at 80 ^o^C for 20 min. Then the reaction mixture was cooled to room temperature. The crude precipitate formed was filtrated, washed with H_2_O (20 mL), dried, and re-crystallized from EtOH. Yield is 83%. The single-crystals of the product I were obtained by slow crystallization from EtOH. *M*.p. = 398-399 K. IR (KBr), ν/cm^-1^: 3301, 1693, 1550, 1495, 1181, 972, 872. ^1^H NMR (600 MHz, DMSO-*d_6_*, 304 K): 2.55 (s, 3H), 4.88 (s, 2H), 7.62 (d, 2H, *J* = 8.8), 7.87 (d, 2H, *J* = 8.8). Anal. Calcd for C_11_H_9_Br_2_N_3_O: C, 42.00; H, 2.88. Found: C, 42.07; H, 2.92.

### S3. Refinement

The absolute configuration of I was objectively determined by the refinement of Flack parameter (3428 (99%) Friedel pairs measured) to 0.032 (7). The calculated Hooft parameter is equal to 0.023 (6).

All hydrogen atoms were placed in the calculated positions with C–H = 0.95 (aryl-H), 0.98 (methyl-H) and 0.99 (methylene-H) Å and refined in the riding model with fixed isotropic displacement parameters: *U*_iso_(H) = 1.2-1.5*U*_eq_(C).

### Crystal data

**Table:**

C_11_H_9_Br_2_N_3_O	*F*(000) = 696
*M**_r_* = 359.03	*D*_x_ = 2.007 Mg m^−^^3^
Monoclinic, *P**n*	Mo *K*α radiation, λ = 0.71073 Å
Hall symbol: P -2yac	Cell parameters from 9920 reflections
*a* = 3.9699 (10) Å	θ = 2.5–32.2°
*b* = 19.437 (5) Å	µ = 6.81 mm^−^^1^
*c* = 15.402 (4) Å	*T* = 100 K
β = 90.908 (3)°	Needle, colourless
*V* = 1188.3 (5) Å^3^	0.30 × 0.03 × 0.03 mm
*Z* = 4	

### Data collection

**Table:**

Bruker APEXII CCD diffractometer	6924 independent reflections
Radiation source: fine-focus sealed tube	6426 reflections with *I* > 2σ(*I*)
Graphite monochromator	*R*_int_ = 0.028
φ and ω scans	θ_max_ = 30.0°, θ_min_ = 2.1°
Absorption correction: multi-scan (*SADABS*; Bruker, 2003)	*h* = −5→5
*T*_min_ = 0.235, *T*_max_ = 0.822	*k* = −26→27
18272 measured reflections	*l* = −21→21

### Refinement

**Table:**

Refinement on *F*^2^	Secondary atom site location: difference Fourier map
Least-squares matrix: full	Hydrogen site location: inferred from neighbouring sites
*R*[*F*^2^ > 2σ(*F*^2^)] = 0.028	H-atom parameters constrained
*wR*(*F*^2^) = 0.067	*w* = 1/[σ^2^(*F*_o_^2^) + (0.0221*P*)^2^ + 0.4369*P*] where *P* = (*F*_o_^2^ + 2*F*_c_^2^)/3
*S* = 1.04	(Δ/σ)_max_ = 0.001
6924 reflections	Δρ_max_ = 0.72 e Å^−^^3^
309 parameters	Δρ_min_ = −0.59 e Å^−^^3^
2 restraints	Absolute structure: Flack (1983), 3428 Friedel pairs
Primary atom site location: structure-invariant direct methods	Absolute structure parameter: 0.032 (7)

### Special details

**Table:**

Geometry. All esds (except the esd in the dihedral angle between two l.s. planes) are estimated using the full covariance matrix. The cell esds are taken into account individually in the estimation of esds in distances, angles and torsion angles; correlations between esds in cell parameters are only used when they are defined by crystal symmetry. An approximate (isotropic) treatment of cell esds is used for estimating esds involving l.s. planes.
Refinement. Refinement of F^2^ against ALL reflections. The weighted R-factor wR and goodness of fit S are based on F^2^, conventional R-factors R are based on F, with F set to zero for negative F^2^. The threshold expression of F^2^ > 2sigma(F^2^) is used only for calculating R-factors(gt) etc. and is not relevant to the choice of reflections for refinement. R-factors based on F^2^ are statistically about twice as large as those based on F, and R- factors based on ALL data will be even larger.

### Fractional atomic coordinates and isotropic or equivalent isotropic displacement parameters (Å^2^)

**Table:**

	*x*	*y*	*z*	*U*_iso_*/*U*_eq_	
Br1	1.07896 (7)	0.863924 (17)	1.13454 (2)	0.02912 (7)	
Br2	0.64876 (8)	0.877518 (19)	0.33776 (2)	0.03474 (8)	
O1	0.8585 (6)	0.94649 (11)	0.50458 (14)	0.0356 (5)	
N1	0.7274 (6)	0.85632 (12)	0.75006 (16)	0.0257 (5)	
N2	0.5627 (7)	0.79965 (16)	0.71724 (18)	0.0297 (6)	
N3	0.5412 (7)	0.80839 (14)	0.63358 (19)	0.0281 (5)	
C4	0.6933 (8)	0.86928 (14)	0.61085 (19)	0.0251 (5)	
C5	0.8151 (7)	0.90045 (14)	0.68650 (18)	0.0250 (5)	
C6	0.8065 (7)	0.85860 (15)	0.84105 (19)	0.0257 (5)	
C7	0.9625 (7)	0.80248 (15)	0.87919 (18)	0.0270 (6)	
H7	1.0134	0.7630	0.8454	0.032*	
C8	1.0442 (8)	0.80396 (16)	0.9666 (2)	0.0290 (6)	
H8	1.1526	0.7658	0.9936	0.035*	
C9	0.9654 (7)	0.86206 (14)	1.01434 (18)	0.0243 (5)	
C10	0.8081 (8)	0.91854 (15)	0.97703 (19)	0.0282 (6)	
H10	0.7562	0.9578	1.0111	0.034*	
C11	0.7278 (8)	0.91690 (15)	0.8897 (2)	0.0281 (6)	
H11	0.6197	0.9551	0.8627	0.034*	
C12	0.7233 (7)	0.89258 (15)	0.52097 (19)	0.0258 (5)	
C13	0.5797 (8)	0.84371 (17)	0.4533 (2)	0.0270 (6)	
H13A	0.3354	0.8377	0.4627	0.032*	
H13B	0.6890	0.7982	0.4598	0.032*	
C14	1.0031 (8)	0.96530 (16)	0.7007 (2)	0.0304 (6)	
H14A	1.1297	0.9626	0.7557	0.046*	
H14B	0.8445	1.0039	0.7027	0.046*	
H14C	1.1598	0.9724	0.6530	0.046*	
Br3	0.55988 (7)	0.633881 (18)	−0.03542 (2)	0.03091 (8)	
Br4	0.09749 (12)	0.62103 (2)	0.75847 (3)	0.04919 (11)	
O2	0.3422 (6)	0.55351 (11)	0.59535 (15)	0.0374 (5)	
N4	0.2428 (7)	0.64530 (13)	0.34827 (17)	0.0287 (5)	
N5	0.0796 (7)	0.70220 (15)	0.3795 (2)	0.0320 (6)	
N6	0.0522 (7)	0.69383 (14)	0.46281 (19)	0.0304 (5)	
C15	0.2015 (8)	0.63260 (15)	0.4854 (2)	0.0264 (6)	
C16	0.3230 (8)	0.60064 (15)	0.41271 (19)	0.0268 (5)	
C17	0.3215 (7)	0.64193 (15)	0.2580 (2)	0.0274 (6)	
C18	0.4783 (8)	0.69686 (15)	0.21998 (19)	0.0281 (6)	
H18	0.5373	0.7362	0.2535	0.034*	
C19	0.5504 (8)	0.69455 (16)	0.1318 (2)	0.0274 (6)	
H19	0.6588	0.7321	0.1044	0.033*	
C20	0.4609 (7)	0.63628 (15)	0.08477 (19)	0.0261 (6)	
C21	0.3038 (7)	0.58060 (15)	0.12237 (19)	0.0278 (6)	
H21	0.2471	0.5412	0.0888	0.033*	
C22	0.2301 (8)	0.58320 (15)	0.21009 (19)	0.0272 (6)	
H22	0.1196	0.5458	0.2373	0.033*	
C23	0.2154 (8)	0.60812 (15)	0.5765 (2)	0.0286 (6)	
C24	0.0729 (9)	0.65717 (17)	0.6432 (2)	0.0287 (6)	
H24A	−0.1653	0.6671	0.6279	0.034*	
H24B	0.1990	0.7011	0.6412	0.034*	
C25	0.5068 (8)	0.53557 (16)	0.4007 (2)	0.0300 (6)	
H25A	0.6422	0.5385	0.3482	0.045*	
H25B	0.3455	0.4976	0.3949	0.045*	
H25C	0.6551	0.5273	0.4511	0.045*	

### Atomic displacement parameters (Å^2^)

**Table:**

	*U*^11^	*U*^22^	*U*^33^	*U*^12^	*U*^13^	*U*^23^
Br1	0.03673 (15)	0.02734 (16)	0.02318 (14)	−0.00060 (12)	−0.00311 (11)	−0.00161 (13)
Br2	0.04801 (19)	0.03298 (17)	0.02315 (15)	−0.00318 (14)	−0.00196 (12)	0.00142 (13)
O1	0.0514 (14)	0.0268 (11)	0.0287 (11)	−0.0091 (10)	−0.0011 (10)	0.0018 (8)
N1	0.0319 (12)	0.0221 (11)	0.0229 (11)	−0.0024 (9)	0.0001 (9)	−0.0005 (9)
N2	0.0389 (14)	0.0273 (15)	0.0229 (13)	−0.0062 (11)	−0.0002 (10)	−0.0017 (11)
N3	0.0363 (13)	0.0253 (13)	0.0226 (11)	−0.0035 (11)	−0.0004 (9)	−0.0014 (11)
C4	0.0283 (13)	0.0211 (13)	0.0257 (13)	0.0016 (10)	−0.0003 (11)	−0.0007 (10)
C5	0.0290 (13)	0.0198 (12)	0.0261 (13)	−0.0008 (10)	−0.0028 (10)	−0.0014 (10)
C6	0.0297 (13)	0.0254 (13)	0.0220 (12)	0.0005 (11)	−0.0024 (11)	−0.0008 (10)
C7	0.0344 (15)	0.0219 (13)	0.0247 (13)	0.0007 (11)	0.0026 (11)	−0.0026 (10)
C8	0.0351 (14)	0.0228 (14)	0.0291 (15)	0.0025 (12)	0.0024 (11)	0.0005 (13)
C9	0.0293 (14)	0.0223 (13)	0.0213 (12)	−0.0028 (10)	−0.0010 (10)	−0.0015 (10)
C10	0.0336 (15)	0.0216 (13)	0.0293 (14)	0.0007 (11)	−0.0020 (12)	−0.0028 (11)
C11	0.0320 (14)	0.0233 (13)	0.0291 (14)	0.0050 (11)	−0.0009 (11)	0.0005 (11)
C12	0.0301 (13)	0.0233 (13)	0.0239 (13)	0.0005 (10)	−0.0027 (10)	−0.0004 (10)
C13	0.0317 (15)	0.0263 (16)	0.0230 (15)	0.0013 (12)	0.0022 (12)	−0.0030 (13)
C14	0.0377 (16)	0.0213 (14)	0.0321 (15)	−0.0044 (12)	−0.0067 (13)	0.0017 (11)
Br3	0.03856 (16)	0.02766 (17)	0.02664 (16)	0.00068 (13)	0.00437 (12)	−0.00241 (13)
Br4	0.0812 (3)	0.0374 (2)	0.02920 (19)	0.00705 (19)	0.00878 (18)	0.00159 (15)
O2	0.0526 (14)	0.0267 (11)	0.0331 (12)	0.0070 (10)	0.0034 (10)	0.0033 (9)
N4	0.0356 (13)	0.0220 (11)	0.0284 (12)	0.0018 (9)	−0.0011 (10)	−0.0014 (9)
N5	0.0417 (15)	0.0234 (14)	0.0309 (15)	0.0055 (11)	−0.0008 (11)	−0.0035 (12)
N6	0.0375 (13)	0.0245 (13)	0.0291 (13)	0.0048 (11)	−0.0032 (10)	−0.0029 (11)
C15	0.0299 (14)	0.0241 (14)	0.0252 (13)	−0.0010 (11)	0.0035 (12)	−0.0016 (10)
C16	0.0305 (14)	0.0222 (13)	0.0279 (13)	−0.0015 (11)	0.0034 (11)	0.0000 (11)
C17	0.0275 (14)	0.0255 (14)	0.0292 (14)	0.0026 (11)	−0.0005 (11)	−0.0028 (11)
C18	0.0335 (15)	0.0225 (13)	0.0282 (14)	−0.0024 (11)	−0.0031 (11)	−0.0016 (11)
C19	0.0340 (14)	0.0202 (13)	0.0279 (14)	−0.0024 (12)	−0.0035 (11)	0.0025 (13)
C20	0.0278 (14)	0.0250 (14)	0.0253 (13)	0.0018 (10)	−0.0006 (11)	−0.0027 (10)
C21	0.0336 (15)	0.0228 (13)	0.0269 (13)	−0.0001 (11)	−0.0003 (11)	−0.0062 (10)
C22	0.0318 (14)	0.0240 (13)	0.0258 (13)	−0.0060 (11)	−0.0002 (11)	−0.0021 (11)
C23	0.0332 (14)	0.0227 (13)	0.0300 (14)	−0.0027 (11)	0.0014 (12)	−0.0037 (11)
C24	0.0369 (15)	0.0241 (15)	0.0251 (15)	−0.0011 (13)	0.0013 (12)	0.0015 (13)
C25	0.0358 (16)	0.0249 (15)	0.0294 (14)	0.0001 (12)	0.0040 (12)	0.0007 (11)

### Geometric parameters (Å, º)

**Table:**

Br1—C9	1.899 (3)	Br3—C20	1.899 (3)
Br2—C13	1.920 (3)	Br4—C24	1.911 (3)
O1—C12	1.206 (4)	O2—C23	1.208 (4)
N1—C5	1.351 (4)	N4—C16	1.353 (4)
N1—N2	1.373 (4)	N4—N5	1.373 (4)
N1—C6	1.432 (4)	N4—C17	1.432 (4)
N2—N3	1.301 (4)	N5—N6	1.299 (4)
N3—C4	1.376 (4)	N6—C15	1.372 (4)
C4—C5	1.393 (4)	C15—C16	1.375 (4)
C4—C12	1.463 (4)	C15—C23	1.482 (4)
C5—C14	1.479 (4)	C16—C25	1.473 (4)
C6—C7	1.381 (4)	C17—C18	1.372 (4)
C6—C11	1.396 (4)	C17—C22	1.404 (4)
C7—C8	1.380 (4)	C18—C19	1.393 (5)
C7—H7	0.9500	C18—H18	0.9500
C8—C9	1.386 (4)	C19—C20	1.388 (4)
C8—H8	0.9500	C19—H19	0.9500
C9—C10	1.384 (4)	C20—C21	1.381 (4)
C10—C11	1.378 (4)	C21—C22	1.388 (4)
C10—H10	0.9500	C21—H21	0.9500
C11—H11	0.9500	C22—H22	0.9500
C12—C13	1.515 (4)	C23—C24	1.516 (4)
C13—H13A	0.9900	C24—H24A	0.9900
C13—H13B	0.9900	C24—H24B	0.9900
C14—H14A	0.9800	C25—H25A	0.9800
C14—H14B	0.9800	C25—H25B	0.9800
C14—H14C	0.9800	C25—H25C	0.9800
			
C5—N1—N2	111.7 (2)	C16—N4—N5	111.5 (3)
C5—N1—C6	129.4 (2)	C16—N4—C17	129.1 (3)
N2—N1—C6	118.7 (2)	N5—N4—C17	119.3 (3)
N3—N2—N1	106.5 (3)	N6—N5—N4	107.0 (3)
N2—N3—C4	110.0 (3)	N5—N6—C15	108.5 (3)
N3—C4—C5	108.0 (3)	N6—C15—C16	109.9 (3)
N3—C4—C12	123.3 (3)	N6—C15—C23	121.9 (3)
C5—C4—C12	128.6 (3)	C16—C15—C23	128.2 (3)
N1—C5—C4	103.9 (2)	N4—C16—C15	103.1 (3)
N1—C5—C14	124.7 (3)	N4—C16—C25	124.8 (3)
C4—C5—C14	131.4 (3)	C15—C16—C25	132.1 (3)
C7—C6—C11	121.0 (3)	C18—C17—C22	121.6 (3)
C7—C6—N1	118.8 (3)	C18—C17—N4	119.1 (3)
C11—C6—N1	120.2 (3)	C22—C17—N4	119.3 (3)
C8—C7—C6	119.7 (3)	C17—C18—C19	119.5 (3)
C8—C7—H7	120.2	C17—C18—H18	120.2
C6—C7—H7	120.2	C19—C18—H18	120.2
C7—C8—C9	118.9 (3)	C20—C19—C18	118.7 (3)
C7—C8—H8	120.6	C20—C19—H19	120.6
C9—C8—H8	120.6	C18—C19—H19	120.6
C10—C9—C8	122.0 (3)	C21—C20—C19	122.3 (3)
C10—C9—Br1	119.2 (2)	C21—C20—Br3	119.4 (2)
C8—C9—Br1	118.7 (2)	C19—C20—Br3	118.3 (2)
C11—C10—C9	118.9 (3)	C20—C21—C22	118.9 (3)
C11—C10—H10	120.6	C20—C21—H21	120.6
C9—C10—H10	120.6	C22—C21—H21	120.6
C10—C11—C6	119.5 (3)	C21—C22—C17	119.0 (3)
C10—C11—H11	120.3	C21—C22—H22	120.5
C6—C11—H11	120.3	C17—C22—H22	120.5
O1—C12—C4	120.7 (3)	O2—C23—C15	121.2 (3)
O1—C12—C13	124.4 (3)	O2—C23—C24	123.3 (3)
C4—C12—C13	114.9 (3)	C15—C23—C24	115.5 (3)
C12—C13—Br2	111.4 (2)	C23—C24—Br4	112.6 (2)
C12—C13—H13A	109.3	C23—C24—H24A	109.1
Br2—C13—H13A	109.3	Br4—C24—H24A	109.1
C12—C13—H13B	109.3	C23—C24—H24B	109.1
Br2—C13—H13B	109.3	Br4—C24—H24B	109.1
H13A—C13—H13B	108.0	H24A—C24—H24B	107.8
C5—C14—H14A	109.5	C16—C25—H25A	109.5
C5—C14—H14B	109.5	C16—C25—H25B	109.5
H14A—C14—H14B	109.5	H25A—C25—H25B	109.5
C5—C14—H14C	109.5	C16—C25—H25C	109.5
H14A—C14—H14C	109.5	H25A—C25—H25C	109.5
H14B—C14—H14C	109.5	H25B—C25—H25C	109.5
			
C5—N1—N2—N3	−1.0 (4)	C16—N4—N5—N6	−0.7 (4)
C6—N1—N2—N3	−176.0 (3)	C17—N4—N5—N6	−177.2 (3)
N1—N2—N3—C4	0.9 (4)	N4—N5—N6—C15	1.0 (4)
N2—N3—C4—C5	−0.5 (4)	N5—N6—C15—C16	−1.0 (4)
N2—N3—C4—C12	177.5 (3)	N5—N6—C15—C23	179.5 (3)
N2—N1—C5—C4	0.7 (3)	N5—N4—C16—C15	0.0 (3)
C6—N1—C5—C4	175.0 (3)	C17—N4—C16—C15	176.2 (3)
N2—N1—C5—C14	−178.3 (3)	N5—N4—C16—C25	−178.4 (3)
C6—N1—C5—C14	−4.0 (5)	C17—N4—C16—C25	−2.2 (5)
N3—C4—C5—N1	−0.2 (3)	N6—C15—C16—N4	0.6 (3)
C12—C4—C5—N1	−178.0 (3)	C23—C15—C16—N4	180.0 (3)
N3—C4—C5—C14	178.8 (3)	N6—C15—C16—C25	178.8 (3)
C12—C4—C5—C14	1.0 (5)	C23—C15—C16—C25	−1.8 (6)
C5—N1—C6—C7	−124.6 (3)	C16—N4—C17—C18	−126.4 (3)
N2—N1—C6—C7	49.3 (4)	N5—N4—C17—C18	49.5 (4)
C5—N1—C6—C11	55.1 (4)	C16—N4—C17—C22	54.9 (4)
N2—N1—C6—C11	−131.0 (3)	N5—N4—C17—C22	−129.2 (3)
C11—C6—C7—C8	−0.4 (5)	C22—C17—C18—C19	−0.2 (5)
N1—C6—C7—C8	179.3 (3)	N4—C17—C18—C19	−178.8 (3)
C6—C7—C8—C9	0.3 (5)	C17—C18—C19—C20	0.0 (5)
C7—C8—C9—C10	−0.1 (5)	C18—C19—C20—C21	−0.2 (5)
C7—C8—C9—Br1	−179.9 (2)	C18—C19—C20—Br3	179.9 (2)
C8—C9—C10—C11	−0.1 (5)	C19—C20—C21—C22	0.6 (5)
Br1—C9—C10—C11	179.7 (2)	Br3—C20—C21—C22	−179.4 (2)
C9—C10—C11—C6	0.0 (5)	C20—C21—C22—C17	−0.8 (4)
C7—C6—C11—C10	0.2 (5)	C18—C17—C22—C21	0.7 (5)
N1—C6—C11—C10	−179.5 (3)	N4—C17—C22—C21	179.3 (3)
N3—C4—C12—O1	179.9 (3)	N6—C15—C23—O2	178.6 (3)
C5—C4—C12—O1	−2.6 (5)	C16—C15—C23—O2	−0.7 (5)
N3—C4—C12—C13	−1.2 (4)	N6—C15—C23—C24	−3.5 (4)
C5—C4—C12—C13	176.3 (3)	C16—C15—C23—C24	177.1 (3)
O1—C12—C13—Br2	1.2 (4)	O2—C23—C24—Br4	−2.8 (4)
C4—C12—C13—Br2	−177.6 (2)	C15—C23—C24—Br4	179.4 (2)

### Hydrogen-bond geometry (Å, º)

**Table:**

*D*—H···*A*	*D*—H	H···*A*	*D*···*A*	*D*—H···*A*
C10—H10···O1^i^	0.95	2.44	3.205 (4)	137
C13—H13*B*···N6^ii^	0.99	2.49	3.467 (4)	170
C21—H21···O2^iii^	0.95	2.45	3.210 (4)	137
C24—H24*B*···N3	0.99	2.49	3.482 (4)	177

Symmetry codes: (i) *x*−1/2, −*y*+2, *z*+1/2; (ii) *x*+1, *y*, *z*; (iii) *x*−1/2, −*y*+1, *z*−1/2.
